# Gut microbiota-driven IL-17A production by hepatic γδ T cells enhances neutrophil defense against systemic *Staphylococcus aureus* infection

**DOI:** 10.1080/21505594.2026.2629132

**Published:** 2026-02-09

**Authors:** Na Pan, Xing Su, Yifei Meng, Yanchen Liang, Xiye Chen, Haochi Zhang, Xiao Wang

**Affiliations:** a Microbiology and Immunology Laboratory, School of Life Sciences, Inner Mongolia University, Hohhot, China; b Institutes of Biomedical Sciences, Inner Mongolia University, Hohhot, China

**Keywords:** *S. aureus*, systemic infection, gut microbiota, neutrophils, IL-17A, γδ T cells

## Abstract

*Staphylococcus aureus* (*S. aureus*) bloodstream infections pose a significant clinical threat, exacerbated by increasing antibiotic resistance and high mortality. While the gut microbiota is recognized as a key modulator of systemic immunity, the mechanisms underlying its protective role against invasive bacterial infections remain incompletely understood. Here, we investigated how gut microbiota influences hepatic immune responses during early *S. aureus* bloodstream infection using animal models. Our findings demonstrate that the gut microbiota exerts a protective effect against systemic *S. aureus* infection. Specifically, commensal microbiota-derived signals prime hepatic γδ T cells for rapid interleukin-17A (IL-17A) production upon bacterial challenge. This microbiota-dependent IL-17A response subsequently promotes neutrophil recruitment to the liver, facilitating bacterial clearance and limiting systemic dissemination. Disruption of the gut microbiota impaired hepatic γδ T cell IL-17A production, reduced neutrophil mobilization, and compromised host resistance to infection. Notably, we found that colonization with the commensal *Limosilactobacillus reuteri* (*L. reuteri)* activates this hepatic γδT17-neutrophil axis, enhancing host defense against *S. aureus* as a mechanism involving indole metabolites. This study reveals a novel gut-liver axis whereby intestinal microbiota orchestrates hepatic γδ T cell function to establish an early immunological barrier against invasive bacterial pathogens, offering potential therapeutic avenues for enhancing host defense against life-threatening *S. aureus* infections.

## Introduction

*Staphylococcus aureus* (*S. aureus*) is a leading cause of bloodstream infection, and its clinical management is increasingly complicated by the rise of drug-resistant variants such as methicillin-resistant *S. aureus* (MRSA) [1]. Despite advances in antimicrobial therapy, mortality rates remain high, particularly in invasive infections [2], highlighting the urgent need for novel strategies to enhance host defense mechanisms.

The intestinal microbiota plays a pivotal role in modulating immune system development and activity, extending its influence beyond the gastrointestinal tract to distant organs [3,4]. Long-distance communication between the gut microbiota and immune cells in distal organs, mediated by circulating bacterial derivatives and metabolites, plays a critical role in protecting the host from severe bacterial infections [5–9]. Growing evidence associates intestinal dysbiosis with heightened vulnerability to systemic bacterial infections and sepsis in clinical settings. Notably, intensive care unit (ICU) patient studies reveal that microbial imbalance correlates with elevated risks of hospital-acquired infections, sepsis-related organ failure, and fatal outcomes [10–13]. Similarly, in murine sepsis models, disruption of the gut microbiota via antibiotic treatment or germ-free (GF) conditions impairs immune defenses, leading to higher pathogen loads and reduced survival rates [7,9,14–16]. While these findings underscore the importance of microbiota-immune interactions in systemic infection resistance, the precise molecular mechanisms remain incompletely characterized.

The liver serves as a critical immune surveillance hub and a “first line of defense” due to its unique anatomical structure, characterized by abundant sinusoids and a density of resident immune cells like Kupffer cells (KCs) and γδ T cells, among others. This positioning allows it to efficiently process circulating pathogens and microbial products originating from the gut, and gut dysbiosis can directly impact the hepatic microenvironment and immune cell function via the portal circulation. Consequently, in the early stages of systemic bacterial infection, circulating pathogens are effectively trapped and cleared in the liver, establishing a pivotal site for a systemic intravascular immune firewall. A key component of this hepatic defense involves neutrophil homing to this highly vascularized organ. Within the liver’s microcirculation, these neutrophils patrol and collaborate with resident liver macrophages (KCs) to establish a defensive barrier that captures and eliminates circulating bacteria [17,18]. Significantly, the gut microbiota profoundly shapes this neutrophil-dependent host defense mechanism [19]. Disruption of gut microbiota leads to defective neutrophil function during infection, ultimately permitting systemic pathogen spread [19]. The development of neutrophils in hematopoietic tissues is modulated by microbial-derived factors that promote the expansion and specialization of myeloid precursor cells [7,19,20]. Following their release into peripheral circulation, neutrophils undergo functional maturation regulated by distal microbial signals. Toll-like receptor (TLR)-MyD88 signaling pathways activated by gut-derived molecular patterns play a central role in this process [8]. The migratory behavior of neutrophils appears to be similarly influenced by commensal microorganisms, as evidenced by altered neutrophil recruitment patterns to infected sites in microbiota-deficient hosts [21–23]. While these findings demonstrate the gut microbiota’s broad impact on neutrophil biology, the specific regulatory mechanisms governing neutrophil mobilization in systemic *S. aureus* infections remain incompletely characterized.

Among hepatic immune cells, γδ T cells represent a unique lymphocyte population that bridges innate and adaptive immunity [24,25]. Unlike conventional αβ T cells, γδ T cells can rapidly produce proinflammatory cytokines, particularly IL-17A, within hours of microbial encounter [26]. Current understanding of IL-17A in infection has primarily focused on adaptive T helper cell 17 (Th17 cells) responses, which typically develop days after pathogen encounter. In contrast, γδ T cells represent an innate source of IL-17A that can respond within hours of infection, making them particularly relevant for early host defense. This rapid response capability suggests γδ T cells may play a crucial role in the critical early phase of systemic infection when the balance between pathogen clearance and immunopathology is determined. In mucosal tissues, γδ T cell-derived IL-17A is essential for neutrophil recruitment during bacterial infections [27–29]. Emerging evidence indicates that gut microbiota can shape hepatic γδ T cell development and function [30], raising the intriguing possibility that microbial signals may prime hepatic γδ T cells for optimal IL-17A production during subsequent bloodstream infection. This represents a significant knowledge gap, as the liver contains one of the largest reservoirs of γδ T cells in the body [30]. Moreover, while gut microbiota has been shown to influence γδ T cell function in intestinal and pulmonary settings [31,32], its impact on hepatic γδ T cells during bloodstream infections remains completely unexplored.

While the broad impact of the gut microbiota on systemic immunity is well-documented, the precise mechanisms by which commensal bacteria regulate *early hepatic immune responses* during bacterial bloodstream infections are not fully understood. This investigation sought to uncover a novel gut-liver axis, positing that gut microbiota primes hepatic γδ T cells to generate IL-17A, consequently enhancing neutrophil recruitment and host resistance to systemic *Staphylococcus aureus* infection. Through the use of animal models, we established that the gut microbiota significantly contributes to host defense against systemic *S. aureus*. Our results demonstrate that commensal microbiota-derived signals activate hepatic γδ T cells to produce IL-17A, which subsequently drives neutrophil migration to the liver and promotes bacterial clearance. Notably, we found that mono-colonization with *L. reuteri* (WX25) specifically primes this hepatic γδT17-neutrophil axis as a mechanism involving indole metabolites, thereby augmenting host defense. In essence, this study delineates a key gut-liver mechanism wherein the intestinal microbiota establishes an immunological firewall through γδ T cell-mediated IL-17A production, limiting invasive bacterial dissemination.

## Material and methods

### Mice

All experimental procedures involving animals followed the guidelines approved by the Ethics Committee of Inner Mongolia University (Approval No. IMU-mouse-2024–147) and adhered to the ARRIVE 2.0 guidelines and international regulations. Specific pathogen-free (SPF) C57BL/6J wild-type (WT) mice (female, 6–8 weeks old, 20–22 g) were purchased from Beijing Sipeifu Biotech Co. (Beijing, China). *Tcrδ*^+/-^ mice with a C57BL/6J background were kindly donated by Dr. Zhinan Yin (Jinan University, Guangzhou, China). *Il17a*^+/-^ mice (genetic background C57BL/6) were from Cyagen Biosciences (Suzhou, China). IL-17A knockout mice (*Il17a*^−/−^) and γδ T cell-deficient (*Tcrδ*^−/−^) mice were generated by crossing *Il17a*^+/-^ and *Tcrδ*^+/-^ mice. Mice were genotyped by PCR analysis of tail DNA. A total of 34 *Il17a*^−/−^ and 61 *Tcrδ*^−/−^ mice were involved in this study. Mouse care and experimental procedures were performed under SPF conditions. All experimental mice were accommodated under controlled environment conditions with humidity maintained at 40–60%, temperature at 20–22°C, and a standard 12-hour light/dark cycle. They had unrestricted access to standard chow and water. The individual mouse was considered the experimental unit. No formal a *priori* sample size calculation was performed. The group size (*n* = 5–10 mice per group) was determined based on similar published studies [18,33] and practical feasibility, aiming to optimize animal numbers. Mice were randomly allocated to the experimental groups. Potential confounders, such as treatment order and cage location, were not controlled for in this study. No inclusion or exclusion criteria were established for animals during the experiment or for data points during analysis.

### Microbiota disruption and reconstitution

Procedures for microbiota depletion and fecal microbiota transplantation (FMT) followed previously established protocols [29,34]. Gut microbiota depletion in SPF mice was achieved by administering a broad-spectrum antibiotic cocktail (ampicillin 1 g/L, neomycin 1 g/L, metronidazole 1 g/L, vancomycin 0.5 g/L) in drinking water for 3 weeks and changed every 3 days. For FMT preparation, fresh fecal pellets were collected from untreated donor mice, pooled, and immediately homogenized in sterile PBS (at a concentration of 0.1 g feces/mL PBS). Following centrifugation of the homogenate, the supernatant was collected for transplantation. Two days post-antibiotic cessation, antibiotic-treated (ABX) mice began receiving daily oral gavage administrations of 200 μL fecal supernatant for 7 consecutive days for microbiota engraftment. Untreated (No ABX) control mice were similarly gavaged with 200 μL of sterile PBS vehicle.

### Preparation of genomic DNA from murine stool samples

Microbiome characterization utilized total DNA obtained from murine stool samples. Sample collection involved using sterilized forceps to retrieve fecal pellets from mice kept in sterile housing conditions; samples were then maintained frozen (−80°C) and dry until processing. DNA extraction from these samples was performed using the PowerLyzer PowerSoil DNA Extraction Kit (MoBio) according to the manufacturer’s instructions [35].

### Fecal bacteria quantification

Fresh fecal pellets from No ABX and ABX mice were collected, weighted, homogenized, and serially diluted with PBS, then the dilutions of each sample were plated onto tryptic soy agar supplemented with 5% sheep’s blood in aerobic and anaerobic environments respectively. After incubation for 24 hours at 37°C, colony forming units (CFUs) were enumerated using a Scan 300 automated colony counter, and the numbers of fecal bacteria colonies were expressed as CFUs/g feces.

To further quantify bacterial loads in the gastrointestinal tract, DNA extracted from fecal pellets was analyzed for 16S ribosomal (r) DNA by real-time RT-PCR [35]. The RT-qPCR reaction was performed using Power SYBR Green PCR Master Mix (Thermo Fisher Scientific) with designated primers 27F (5’-ACTCCTACGGGAGGCAGCAGT-3’) and 1492 R (5’-ATTACCGCGGCTGCTGGC-3’). Quantitative PCR was performed on an Applied Biosystems StepOnePlus instrument and cycled 40 times with 15 s at 95°C and 1 min at annealing temp. The 16S rRNA gene copy number per gram stool was then calculated using the standard curve.

### Bacterial culture conditions and infections

*S. aureus* strain USA300 was purchased from American Type Culture Collection (Manassas, VA, USA). The *S. aureus* strain USA300 was streaked onto Luria-Bertani (LB) plates and grown at 37°C under aerobic conditions overnight. Single colonies were cultured in LB broth at 37°C in a shaking incubator (220 rpm) for 12 hours. For infections, the overnight culture was sub-cultured until it reached exponential phase (OD_600_ = 1), centrifuged, washed and resuspended in sterile phosphate-buffered saline (PBS) at a concentration of 2 × 10^9^ CFUs/mL. Animals were intravenously (*i.v*.) injected via the tail vein with single injection of *S. aureus* strain USA300. The infection dose used for survival rate assessment was 4 × 10^7^ CFU per mouse, while the dose used for other experiments, such as monitoring body weight changes and determining bacterial burden, was 2 × 10^7^ CFU per mouse. Following systemic *S. aureus* infection, mice were monitored for anticipated adverse events including clinical signs (weight loss, hunched posture, lethargy, respiratory distress, hypothermia) and reduced food/water intake, as well as any unexpected events. Mice meeting predefined humane endpoints, such as sustained body weight loss of ≥20% (persisting > 24 hours) or severe distress, were humanely euthanized, and all adverse events and mortalities were recorded. A *S. aureus*-green fluorescent protein (GFP) mutant strain was constructed by transforming *S. aureus* USA300 with the pCM29 plasmid to constitutively express high levels of GFP [36]. *S. aureus*-GFP were grown in LB containing chloramphenicol (10 μg/mL). The collection, processing, and infection dosage of the *S. aureus*-GFP strain were the same as described above.

### Bacteriological analysis of tissues

Mice were euthanized with 3% isoflurane and disinfected with 70% ethanol. Whole blood was obtained by cardiac puncture using a heparinized syringe. Samples were then centrifuged at 400 × *g* for 20 min for serum retrieval. Alanine aminotransferase (ALT) and aspartate aminotransferase (AST) were analyzed using commercial ELISA kits (Abcam, UK) according to the manufacture’s protocols.

Next, the animals were euthanized by cervical dislocation, and organs were harvested. Specifically, livers, kidneys, spleens, and intestines were collected. The intestines were flushed with PBS to remove their luminal contents. The organs were harvested, weighed, and homogenized in sterile PBS (1 mL/0.1 g tissue) using a tissue homogenizer (Analytikjena, Yena, Germany). Tissue homogenates were prepared in PBS, and serial dilutions were planted on mannitol salt agar (MSA) plates (HuanKai Microbial, Guangzhou, China) and inoculated overnight at 37°C, and bacterial colonies were counted using a Scan 300 automated colony counter.

### Histopathology

The *S. aureus*-infected mice were sacrificed on day 10 of the infection. The livers were fixed in 10% formalin and embedded in paraffin and 4 μm sections were cut and stained with hematoxylin and eosin (H&E).

### Bacterial *in* *vivo* fluorescence imaging

Mice were euthanized under deep anesthesia at 12 hours post-infection with *S. aureus*-GFP, and major organs (liver, spleen, kidney, and lungs) were harvested for fluorescence imaging using an IVIS Lumina III imaging system (PerkinElmer, Waltham, MA, USA) with excitation/emission filters set at 490/520 nm. Fluorescence intensity (radiance, p s^−1^ cm^−2^ sr^−1^) was quantified using Living Image software (v4.7.3) and normalized to background values from uninfected control tissues.

### Flow cytometry

Livers were harvested and cut into small pieces, digested at 37°C for 1 h with a concentration of 1 mg/mL collagenase IV (Worthington Biochemical Corporation, Freehold, NJ, USA). The large pieces of liver were through a 200-mesh steel mesh then red blood cell lysis (RBC) lysed, and the cells washed with PBS. Mononuclear cells (MNCs) were isolated from the flow-through obtained by gradient centrifugation with 42 and 70% Percoll (Solarbio, Beijing, China). FcgRIII/II of MNCs were blocked with an anti-CD16/CD32 mAb (clone 93, Biolegend, 101,302). Cells were stained with fluorochrome-conjugated antibodies (1:100 dilution each), including anti-CD45.2-PerCP-Cy5.5 (clone 104, Invitrogen, 45–0454-82), anti-CD3ε-APC (clone 145-2C11, Invitrogen, 17–0031-83), anti-CD3ε-FITC (clone 145-2C11, Invitrogen, 11–0031-82), anti-F4/80-PE (clone BM8, Biolegend, 123110), anti-CD11b-APC-Cy7 (clone M1/70, Invitrogen, A15390), anti-Ly-6G-PE (clone 1A8, Biolegend, 127608), anti-CD4-PE (clone RM4-5, Invitrogen, 12–0042-82), and anti-TCRγ/δ-APC (clone GL3, Biolegend, 118116). For intracellular cytokine staining, cells were pre-incubated with 50 ng/mL PMA (Sigma), 1 μg/mL ionomycin (Sigma-Aldrich, St. Louis, Mo, USA) and 10 ng/mL Brefeldin A for 5 h at 37°C, 5% CO_2_ before surface staining. After cell surface staining, cells were fixed and permeabilized with Perm/Wash solution (BD Bioscience; San Jose, CA, USA), and separately stained intracellularly with anti-IL-17A-PE-Cy7 (clone eBio17B7, Invitrogen, 25–7177-82) or anti-IL-17A-PE (clone eBio17B7, Invitrogen, 12–7177-81). Flow cytometry (FCM) was performed based on the NovoCyte 2060 R (ACEA), and data were analyzed using software program NovoExpress (ACEA).

### ELISA for cytokine level

Liver tissue homogenates were obtained as above, and the expression level of IL-17A, KC (CXCL-1), MIP-2 (CXCL-2), and myeloperoxidase (MPO) were measured by enzyme-linked immunosorbent assay (ELISA) kit (R&D Systems, Minneapolis, MN, USA) according to the manufacture’s protocols.

### Administration of rIL-17A

A single dose of recombinant IL-17A (rIL-17A; MCE), at a dose of 1 μg per mouse based on previous studies [27,28], was dissolved in sterile PBS. rIL-17A or vehicle were mixed with the inoculum of *S. aureus* and administered intradermally to *Tcrδ*^−/−^ mice.

### 16S amplicon sequencing and analysis

16S rRNA sequencing libraries were prepared based on a previously described strategy [29]. The V4 region of the 16S rRNA gene was amplified using primers 515F (5”-GTGYCAGCMGCCGCGGTAA-3‘) and 806 R (5’-GGACTACNVGGGTWTCTAAT-3‘) with barcodes. All PCR reactions were purified using the Qiagen Gel Extraction Kit (Qiagen, Germany). Sequencing libraries were generated using the TruSeq DNA PCR-Free Sample Preparation Kit (Illumina, USA) following the manufacturer’s recommendations and index codes were added. Library quality was assessed on the Qubit @ 2.0 Fluorometer (Thermo Fisher Scientific) and Agilent Bioanalyzer 2100 system. Finally, the library was sequenced on aNovoSeq 6000 platform, and 250-bp paired-end reads were generated.

### Colony isolation and identification

Stools from SPF mice were transferred into a pre-tarred tube with 1 mL of sterile PBS. Stool samples were homogenized using homogenizer (Analytikjena, Yena, Germany), serially diluted in sterile PBS and plated onto LB and Man, Rogosa, and Sharpe (MRS; Huankai Microbial Sci. & Tech., Guangdong, China) agar overnight at 37°C in container with ambient air (aerobic) or in Whitley DG250 Anaerobic Workstation (Don Whitley Scientific Limited, Shipley, UK). Agar plates were inspected for colonies exhibiting distinct morphologies. From three independent experiments, over 13 unique colonies were selected for isolation, ensuring purity through subsequent re-streaking to confirm derivation from a single colony. For each confirmed isolate, the full-length 16S rRNA gene was targeted for amplification via PCR using primers 27F (5′-AGAGTTTGATCMTGGCTCAG-3′) and 1492 R (5′-GGTTACCTTGTTACGACTT-3′) at 500 nM each. The thermal cycling parameters were: an initial 3-min denaturation at 98°C; 34 cycles consisting of 30 s denaturation at 98°C, 30 s annealing at 53°C, and 30 s extension at 72°C; followed by a final 3-min extension at 72°C. Column-purified (Qiagen) amplicons were subjected to Sanger sequencing across the full length. A consensus sequence was determined for each isolate, and taxonomic identification was achieved via BLAST comparison against the NCBI 16S rRNA gene reference database (requiring 98–100% sequence identity).

### Administration of *Lactobacillus*

Overnight anaerobic cultures of *Lactobacillus* strains grown in 5 mL of MRS broth (Huankai, China) were washed and diluted with sterile PBS. A final dose of 1 × 10^9^ CFU bacteria in 200 μL PBS per mouse was orally gavaged daily for 10 days. For each administration, CFU doses were confirmed by serial dilution and plating.

### Kovac’s reagent assay

*L. reuteri* WX25, WX26; *L. plantarum* WX27, WX28; *L. murine* WX29; *L. intestinalis* WX30; *E. faecium* WX31, WX32; *E. faecalis* WX33; *B. caecimuris* WX34 isolated from mouse intestine were cultured anaerobically in MRS broth at 37°C for 24 hours. *E. coli* ATCC 25922 (positive control) and *S. typhimurium* ATCC 14028 (negative control) were cultured aerobically in LB broth at 37°C for 24 hours. Bacteria were collected by centrifugation at 5000 × *g* for 10 min, washed by PBS, resuspended in peptone-tryptone-tryptophan (PTT) medium, and cultured anaerobically at 37°C, shaking at 250 rpm, for 14 h. Aliquoted 200 μL of PTT medium, which bacteria has been removed by centrifugation, added 50 μL of Kovac’s regent (Thermo Fisher Scientific), and shaken gently. Observe for the development of a color change, manifested as a ring at the interface between the broth and the solvent.

### Administration of indole metabolites

For indoles supplementation, ABX-treated mice were oral gavaged daily with 30 mg/kg body weight mixture indoles (IAld + IAA + IPA; Coolaber; mixed at 1:1:1) or single component for 10 days.

### Quantification and statistical analysis

Group allocation and experimental procedures were unblinded to investigators and participants. However, outcome assessment and data analysis were performed by blinded assessors and statisticians until completion. Statistical detail is indicated in the figure legends. Data were analyzed using software program GraphPad Prism V. 10.4.1 (San Diego, CA, USA). Values are expressed as mean ± SD. Unpaired t-test or one-way analysis of variance (ANOVA) was used for comparison of two group or multiple groups. A non-parametric test analysis (Kruskal-Walis or Mann-Whitney) was used for comparison groups with few samples. *, *p* < 0.05; **, *p* < 0.01; ***, *p* < 0.001. Data are presented as mean ± SD.

## Results

### Gut microbiome disruption increases host susceptibility to systemic *S.* *aureus* infection

**Figure 1.**
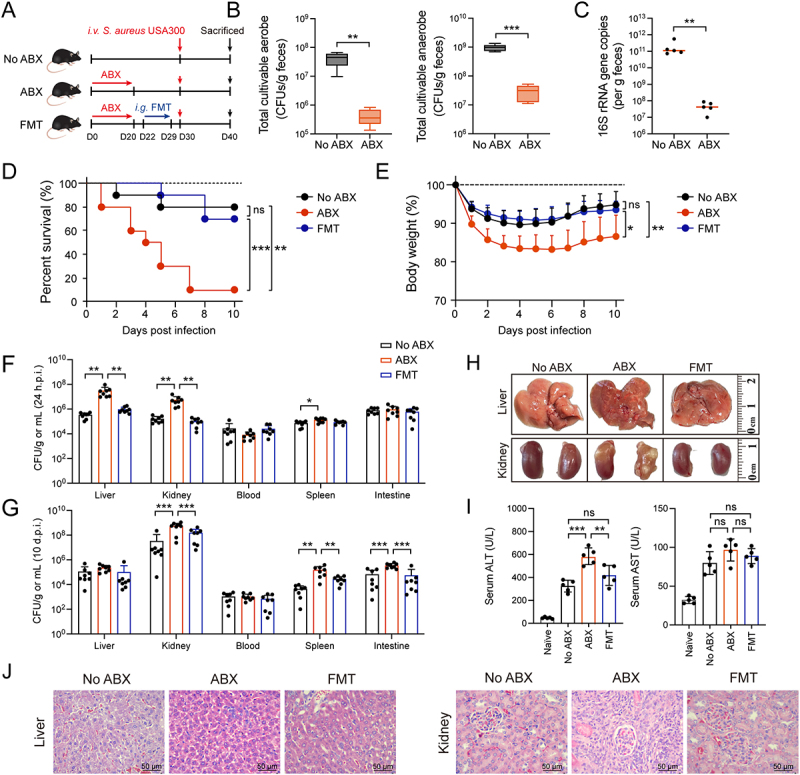
Gut microbiome disruption increases host susceptibility to systemic *S. aureus* infection. (A) This experimental aimed to investigate the role of the gut microbiota in host susceptibility to systemic *S. aureus* infection. The experimental design involved three groups: control (No ABX), ABX (antibiotic-drinking for microbiota depletion), and FMT (for microbiota reconstitution). Subsequently, all mice were challenged *i.v*. with *S. aureus* USA300. (B) Quantification of cultivable aerobes and anaerobes in fecal samples from No ABX and ABX mice (*n* = 5). (C) Quantification of 16S rRNA gene copies in fecal samples from No ABX and ABX mice, measured by RT-PCR (*n* = 5). (D) survival curves of No ABX, ABX, and FMT mice challenged with 4 × 10^7^ CFUs *S. aureus* (*n* = 10). Statistical significance was determined by the log-rank (mantel-cox) test. (E) Body weight loss in No ABX, ABX, and FMT mice challenged with 2 × 10^7^ CFUs *S. aureus* (*n* = 7). (F, G) Bacterial burden in livers, kidneys, spleens, intestines, and blood from No ABX, ABX, and FMT mice at 24 hours and 10 days post-infection (*n* = 8). (H) Representative macroscopic images of the livers and kidneys 10 days post-infection. (I) serum ALT and AST level of No ABX, ABX, and FMT mice at 24 hours post-infection (*n* = 5). (J) Representative images of liver and kidney by H&E staining at 24 hours post-infection. Bar: 50 μm. Data are presented as means ± SD. **p < 0.01; ***p < 0.001; ns, not significant; one-way ANOVA with Tukey’s for multiple comparisons.

To investigate the role of gut microbiota in mediating host defenses against invasive *S. aureus*, we examined the effect of antibiotic-induced gut microbiota disruption on host susceptibility to systemic *S. aureu*s infection. SPF WT mice received at least 21 days of pretreatment with a broad-spectrum antibiotic cocktail (ampicillin, metronidazole, neomycin, and vancomycin) to disrupt their gut microbiota (Figure 1A). All mice were then challenged *i.v*. with 2 × 10^7^ to 4 × 10^7^ CFUs of *S. aureus* USA300. Subsequently, mouse survival rates, body weight loss, bacterial burden, and histopathological changes in the liver and kidneys were assessed. We confirmed that ABX treatment significantly reduced the quantification of total cultivable aerobes and anaerobes (Figure 1B), and 16S rRNA gene copy numbers showed consistent results (Figure 1C). Animals treated with ABX or FMT did not exhibit significant changes in body weight or serum ALT levels (data not shown). ABX mice exhibited a significantly reduced survival rate of 10% after challenge compared with No ABX mice (Figure 1D). Furthermore, ABX mice showed greater body weight loss than No ABX mice, reaching a maximum decline of approximately 20% from baseline (Figure 1E). Two days after cessation of antibiotic treatment, mice were received FMT via oral gavage to reconstitute their microbiota (Figure 1A). Compared with ABX mice, FMT mice showed significantly improved survival rates and reduced weight loss (Figure 1D,E). At 24 hours post-infection, ABX mice had significantly higher bacterial burdens in the liver and kidneys than No ABX and FMT mice (Figure 1F). By day 10 post-infection, these differences were observed in the kidneys, spleens, and intestines (Figure 1G). Existing evidence suggests that during the early phase of systemic *S. aureus* infection, the liver – an organ enriched with innate immune cells – acts as a critical immunological firewall to prevent pathogen dissemination to peripheral tissues. Notably, the gut microbiota actively enhances hepatic immunity against systemic *S. aureus* infection. In addition, the severity of abscess formation in the liver and kidneys, the extent of liver damage, and the histopathological changes in the liver and kidneys in ABX mice were significantly more pronounced than those in No ABX and FMT mice (Figure 1H–J). These results indicate that the gut microbiota promotes host defense against systemic *S. aureus* infection.

### Gut microbiota promote neutrophil trafficking to the liver during early stages of *S.* *aureus* infection

**Figure 2.**
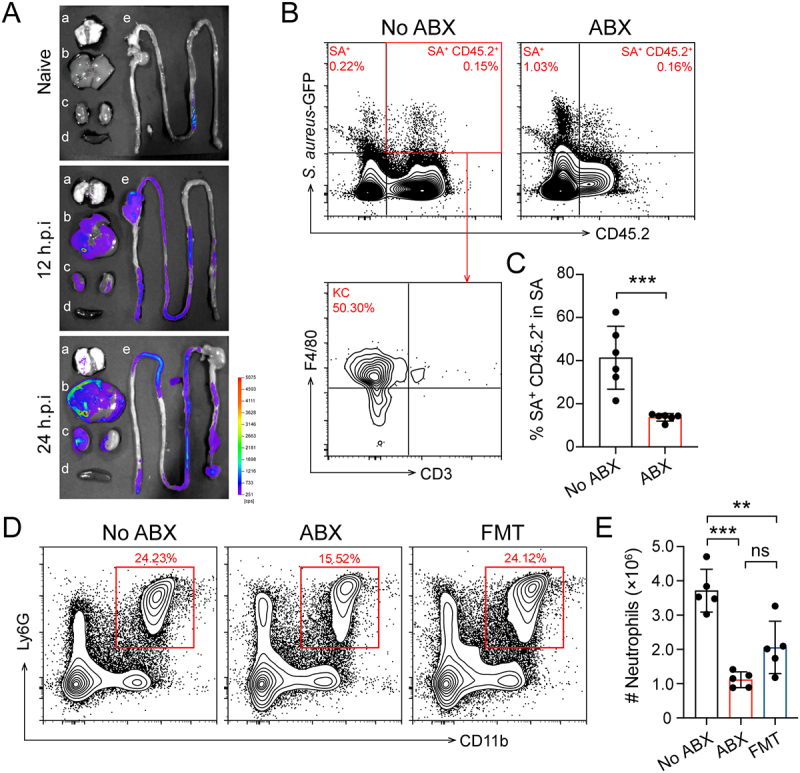
Gut microbiota regulated neutrophils recruitment to liver to mediate hose defense during early infection. This experimental aimed to explore the impact of the gut microbiota on the hepatic immune defense during the early stages of systemic *S. aureus* infection. The experimental design included No ABX, ABX, and FMT groups. (A) WT mice were *i.v*. infected with *S. aureu*s-GFP. Bacterial dissemination was tracked at 12 and 24 hours post-infection using an *in vivo* imaging system (IVIS). Representative images are shown. a: lung; b: liver; c: kidney; d: kidney; e: intestine. (B) No ABX and ABX mice were *i.v*. infected with *S. aureu*s-GFP. The percentage of *S. aureus* associated with hepatic immune cells was determined by FCM by gating on GFP^+^ CD45.2^+^ events. (C) scatter plots shown frequency of *S. aureus*-GFP^+^ CD45.2^+^ in liver at 12 hours post-infection (*n* = 6). (D) Representative contour plots of neutrophils (CD11b^+^ Ly6G^+^) in liver from No ABX, ABX, and FMT mice at 12 hours post-infection. (E) scatter plots shown absolute number of neutrophils in liver at 12 hours post-infection (*n* = 5). Data are presented as means ± SD. *p < 0.05; ***p < 0.001; unpaired *t*-test for two groups comparisons; one-way ANOVA with Tukey’s for multiple comparisons.

To further investigate the dissemination of *S. aureus* in various organs during early infection, WT mice were *i.v*. infected with a *S. aureus*-GFP strain. This strain was engineered to express GFP, enabling tissue and histological bacterial imaging [36]. Using *ex vivo* imaging, colonization and dissemination of *S.* *aureus* were tracked, with the primary site of infection localized to the liver at 12 and 24 hours post-infection (Figure 2A). These *ex vivo* tissue imaging results were consistent with the bacterial load of *S. aureus* observed in organs during the early stages of infection. To investigate the role of the gut microbiota in regulating hepatic immune defense against bacterial infection during the early phase, we infected No ABX and ABX mice. FCM analysis revealed that approximately 40% of hepatic *S. aureus* was captured by CD45^+^ immune cells (predominantly Kupffer cells) in No ABX mice, while this was significantly reduced in ABX mice (Figure 2B,C). In addition, ABX mice exhibited markedly reduced neutrophil recruitment to the liver compared with both No ABX and FMT mice at 12 hours post-infection (Figure 2D,E). These results demonstrate that *S. aureus* predominantly accumulates in the liver during early infection, and that the gut microbiota plays a crucial role in enhancing host defense by promoting neutrophil trafficking to the liver.

### Gut microbiota drive neutrophil recruitment via a IL-17A dependent pathway

**Figure 3.**
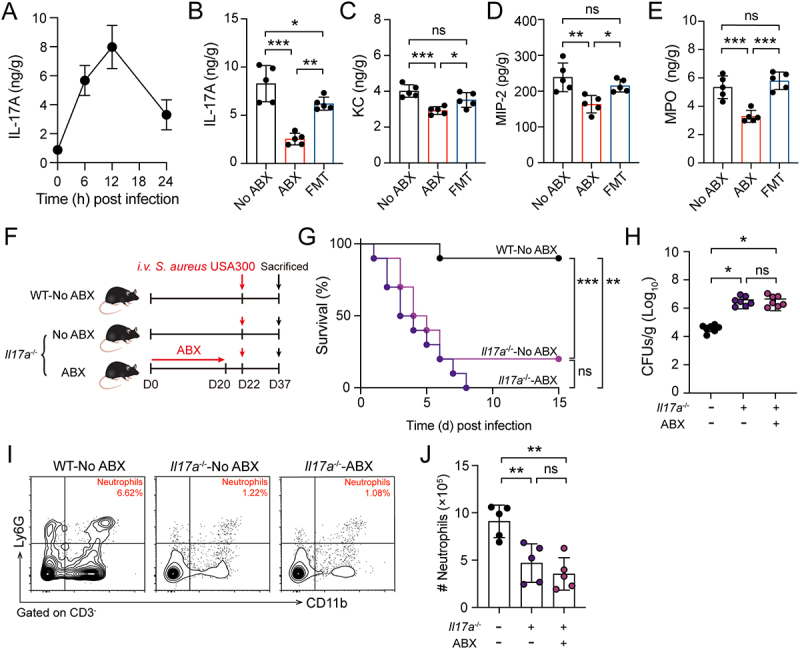
Gut microbiota drive neutrophil recruitment via a IL-17A dependent pathway. The experimental aimed to explore the role of IL-17A in the gut microbiota-mediated enhancement of host resistance against systemic *S. aureus* infection. The experimental utilized WT mice assigned to No ABX, ABX, and FMT groups, as well as *Il17a*^−/−^ mice assigned to No ABX and ABX groups. (A) WT mice were *i.v*. injected with *S. aureus* USA300, the level of IL-17A was determined in liver tissues by ELISA at 0, 6, 12, and 24 hours post-infection (*n* = 5). (B-E) Hepatic IL-17A, KC, MIP-2, and MPO levels in No ABX, ABX, and FMT mice were measured by ELISA at 12 hours post-infection (*n* = 5). (F) Schematic shows that *Il17a*^−/−^ mice were pretreated with either No ABX or ABX containing water. Subsequently, along with the WT-No ABX group, all mice were challenged *i.v*. with *S. aureus* USA300. (G) Survival curves of WT-No ABX, *Il17a*^−/−^-No ABX, and *Il17a*^−/−^- ABX mice after *i.v*. challenged with *S. aureus* (*n* = 10). (H) Bacterial burden in livers of WT-No ABX, *Il17a*^−/−^-No ABX, and *Il17a*^−/−^- ABX mice at 12 hours post-infection (*n* = 7). (I) Representative contour plots of neutrophils (CD3^−^ CD11b^+^ Ly6G^+^) in liver at 12 hours post-infection. (J) Scatter plots shown absolute number of neutrophils in liver at 12 hours post-infection. (*n* = 5). Data are presented as means ± SD. *p < 0.05; **p < 0.01; ***p < 0.001; ns, not significant; one-way ANOVA with Tukey’s for multiple comparisons.

Regulation of IL-17A is a key mechanism controlling granulocytosis and neutrophil recruitment during bacterial infection [7,27]. The dynamics of hepatic IL-17A levels in response to systemic *S. aureus* infection were meticulously examined, and IL-17A levels peaked at 12 hours post-infection (Figure 3A). Compared with No ABX mice, ABX mice exhibited significantly reduced hepatic IL-17A production, which was restored in FMT mice (Figure 3B). IL-17A regulates the production of CXC chemokines such as KC/CXCL1 and MIP-2/CXCL2, as well as MPO, which are necessary for neutrophil recruitment [37]. The hepatic levels of KC, MIP-2, and MPO in ABX mice were lower than those in No ABX and FMT mice (Figure 3C–E). To determine whether IL-17A is a critical mediator of the gut microbiota’s protective role against systemic *S. aureus* infection, *Il17a*^−/−^ mice were pretreated with either No ABX or ABX-containing water (Figure 3F). Both No ABX and ABX-treated *Il17a*^−/−^ mice showed markedly lower survival rates and higher hepatic bacterial burden compared with WT mice; there was no significant difference between No ABX and ABX-treated *Il17a*^−/−^ mice (Figure 3G,H). These results suggest that IL-17A is critical for host resistance to systemic *S. aureus* infection, and the process is mediated by gut microbiota. Furthermore, neutrophil levels in liver were examined by FCM based on CD11b and Ly-6 G co-expression, and it was found that the absence of IL-17A led to a significant reduction in neutrophil infiltration in the liver (Figure 3I,J). These findings demonstrate that the gut microbiota recruits neutrophils to the liver via IL-17A, thereby facilitating bacterial clearance and enhancing host resistance to systemic *S. aureus* infection.

### Gut microbiota induces IL-17A production by hepatic γδ T cells to promote host systemic defense

**Figure 4.**
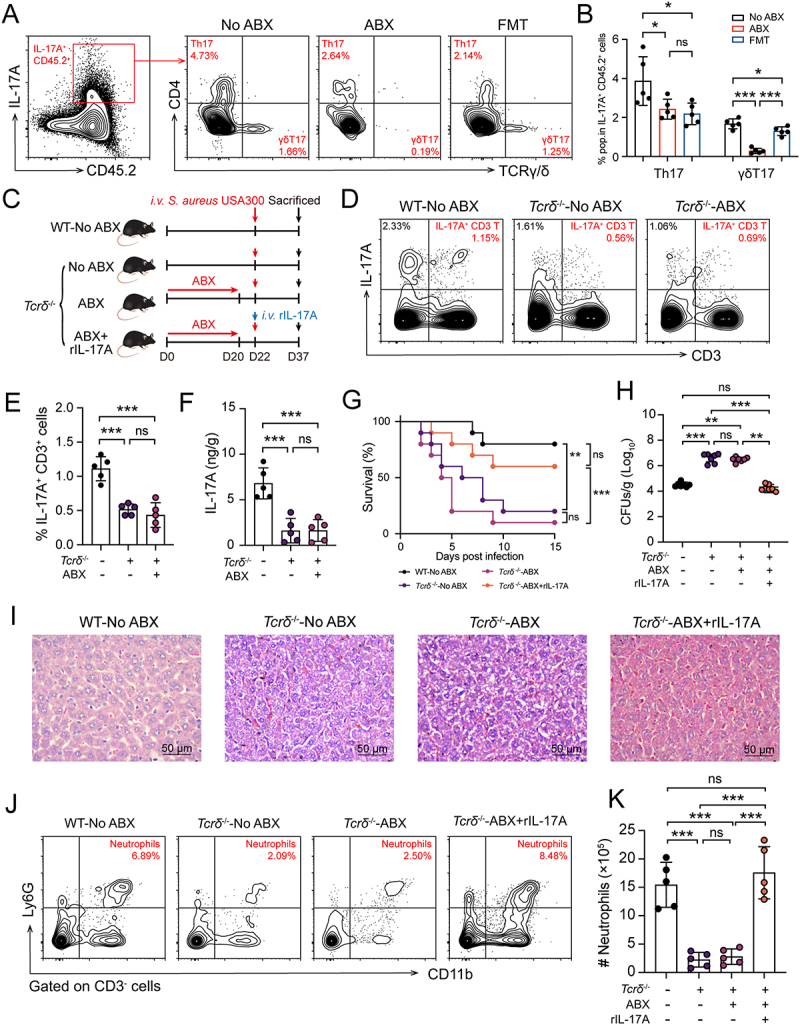
Gut microbiota induces IL-17A production by hepatic γδ T cells to promote host systemic defense. The experimental aimed to identify the cellular sources of the key immune factor IL-17A in the liver during gut microbiota-mediated host defense against systemic *S. aureus* infection. The experimental utilized WT mice assigned to No ABX, ABX, and FMT groups, as well as *Tcrδ*^−/−^ mice assigned to No ABX, ABX, and ABX+rIL-17A groups. (A) No ABX, ABX, and FMT mice were *i.v*. challenged with *S. aureus* USA300, and hepatic Th17 and γδT17 cell levels were measured at 12 hours post-infection. Representative contour plots of hepatic Th17 and γδT17 cells. (B) Scatter plots shown frequencies of hepatic Th17 and γδT17 cells (*n* = 5). (C) Schematic shows that *Tcrδ*^−/−^ mice were pretreated with either No ABX or ABX containing water, or administration with rIL-17A. Subsequently, along with the WT-No ABX group, all mice were challenged *i.v*. with *S. aureus* USA300. (D) The level of IL-17A^+^ CD3^+^ cells in liver were measured in WT-No ABX, *Tcrδ*^−/−^-No ABX, and *Tcrδ*^−/−^-ABX mice at 12 hours post-infection (*n* = 5). Representative contour plots of hepatic IL-17A^+^ CD3^+^ cells. (E) Scatter plots shown frequencies of hepatic IL-17A^+^ CD3^+^ cells. (F) ELSIA analyze level of IL-17A in liver. (G) Survival curves of WT-No ABX, *Tcrδ*^−/−^-No ABX, *Tcrδ*^−/−^-ABX, and *Tcrδ*^−/−^-ABX+rIL-17A mice after *i.v*. challenged with *S. aureus* (*n* = 10). Statistical significance was determined by the log-rank (mantel-cox) test. (H) Bacterial burden in livers from WT-No ABX, *Tcrδ*^−/−^-No ABX, *Tcrδ*^−/−^-ABX, and *Tcrδ*^−/−^-ABX+rIL-17A mice at 12 hours post-infection (*n* = 7). (I) Representative images of liver and kidney by H&E staining. Bar: 50 μm. (J) Representative contour plots of neutrophils in liver at 12 hours post-infection. (K) scatter plots shown absolute number of neutrophils in liver at 12 hours post-infection. Data are presented as means ± SD. *p < 0.05; **p < 0.01; ***p < 0.001; ns, not significant; one-way ANOVA with Tukey’s for multiple comparisons.

To investigate the cellular sources of IL-17A production promoted by gut microbiota in the process of host systemic defense, we compared Th17 (CD3^+^ CD4+ IL-17A^+^) cells and γδT17 (CD3^+^ TCRγ/δ^+^ IL-17A^+^) cells in the livers of No ABX, ABX, and FMT mice. The results showed a significant reduction in γδT17 cells in the livers of ABX mice, while no significant changes were observed in Th17 cells (Figure 4A,B), suggesting that the gut microbiota may mediate immune protection by promoting hepatic γδT17 cells. To validate this hypothesis, we administered No ABX and ABX-treated water to *Tcrδ*^−/−^ mice, followed by systemic infection with *S. aureus* USA300 (Figure 4C). The results showed that a significant decrease in IL-17A^+^ CD3^+^ cells in the livers of *Tcrδ*^−/−^ mice compared with WT-No ABX mice, and there was no significant difference between No ABX and ABX-treated *Tcrδ*^−/−^ mice (Figure 4D,E). Consistently, ELISA confirmed lower hepatic IL-17A levels in *Tcrδ*^−/−^ mice (Figure 4F). Challenge experiments demonstrated that *Tcrδ*^−/−^ mice (regardless of antibiotic treatment) exhibited significantly lower survival rates, higher liver bacterial loads, and more severe liver pathological damage compared to WT mice (Figure 4G–I). Recombinant IL-17A (rIL-17A) supplementation markedly improved survival, reduced bacterial burden, and alleviated liver damage in *Tcrδ*^−/−^ mice (Figure 4C, G–I). Furthermore, the number of neutrophils in the livers of *Tcrδ*^−/−^ mice was significantly lower than that in WT mice and rIL-17A-supplemented *Tcrδ*^−/−^ mice (Figure 4J,K). These data suggest that the gut microbiota promotes IL-17A production by hepatic γδ T cells, mediating neutrophil recruitment to the liver, facilitating bacterial clearance and enhancing host resistance to systemic *S. aureus* infection.

### Gram-positive bacterial disruption exacerbates host susceptibility to systemic *S.* *aureus* infection

**Figure 5.**
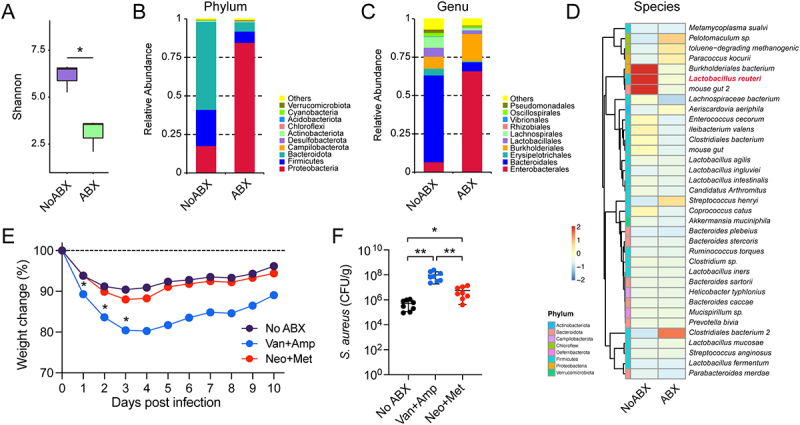
Gram-positive bacterial disruption exacerbates host susceptibility to systemic *S. aureus* infection.infection. The experimental aimed to characterize the effect of antibiotic treatment on the gut microbiome, and preliminarily identify the key bacterial taxa mediating the protective effect. The experimental design included No ABX, ABX, Van+Amp, and Neo+Met groups. (A) α-diversity evaluation of microbial richness evenness of gut microbiome obtained from 16S rDNA sequencing of the feces of No ABX and ABX mice by measuring Shannon diversity indexes. (B, C) Community barplot analysis of relative abundance at the phylum and genus level in feces from No ABX and ABX mice. (D) Heatmap of species-level gut microbiota profiles from feces of No ABX and ABX mice. (*n* = 3). (E) Body weight loss of No ABX, Van+Amp, Neo+Met mice after *i.v*. challenged with *S. aureus* (*n* = 10). (F) Bacterial burden in livers at 12 hours post-infection (*n* = 8). *p < 0.05; **p < 0.01; unpaired *t*-test for two groups comparisons; one-way ANOVA with Tukey’s for multiple comparisons.

To characterize the gut microbiome following antibiotic treatment, 16S rRNA amplicon sequencing was performed on fecal samples from No ABX and ABX mice. The results revealed a significant decrease in gut microbial diversity following ABX treatment (Figure 5A), with marked compositional shifts. At the phylum level, the relative abundance of Firmicutes and Bacteroidota was decreased after antibiotic treatment; at the genus level, the relative abundance of Lactobacillales, Lachnospirales, Bacteroidales was decreased (Figure 5B,C). Furthermore, species-level analysis identified *L. reuteri* as one of the most significantly decreased strains (Figure 5D). Next, we tried to identify the commensal bacteria responsible for the protection against *S. aureus* infection, we utilized the combination of Van with Amp to clear Gram-positive bacteria or the combination of Neo with Met to deplete anaerobic and Gram-negative bacteria [38,39], before inducing systemic *S. aureus* infection. The results showed that Van+Amp-treated mice exhibited the most severe weight loss and significantly higher bacterial burdens compared to No ABX mice, while Neo+Met-treated mice displayed milder symptoms (Figure 5E,F). These findings suggest that Gram-positive bacteria in the gut microbiota may mediate the observed protective effect.

### Commensal *Limosilactobacillus reuteri* activates hepatic γδT17-neutrophil axis to promote host defense to systemic *S.* *aureus* infection

**Figure 6.**
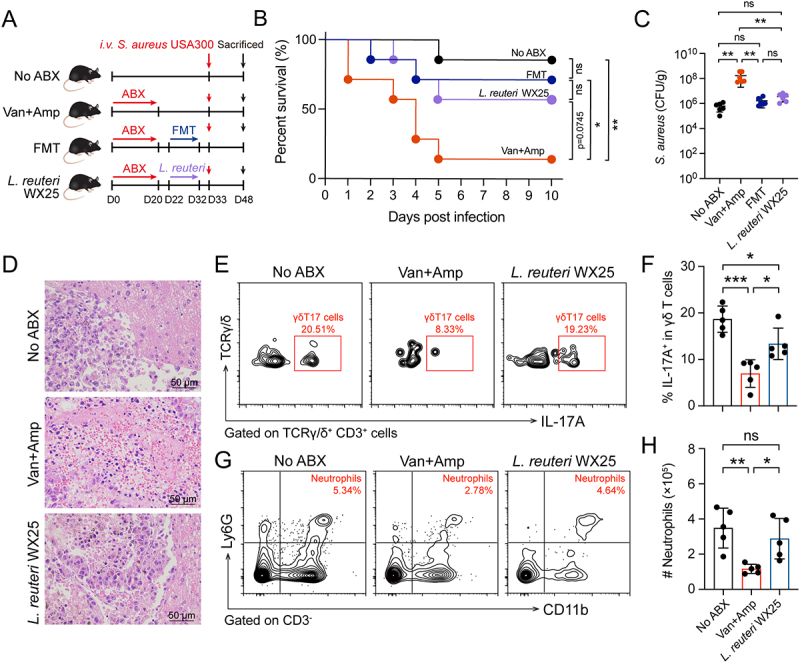
Gut microbiota induces IL-17A production by hepatic γδ T cells to promote host systemic defense. (A) The experimental aimed to determine the contribution of *L. reuteri* WX25, a specific commensal strain derived from the gut microbiota of healthy mice, to host protection against systemic *S. aureus* infection. The experimental design included the No ABX, Van+Amp, Van+Amp+FMT, and Van+Amp+*L. reuteri* WX25 groups. Subsequently, all mice were challenged *i.v*. with *S. aureus* USA300. (B) Survival curves of No ABX, Van+Amp, FMT, *L. reuteri* WX25 mice after *i.v*. challenged with *S. aureus* (*n* = 10). Statistical significance was determined by the log-rank (mantel-cox) test. (C) bacterial burden in livers from No ABX, Van+Amp, Van+Amp+FMT, and Van+Amp+*L. reuteri* WX25 groups at 12 hours post-infection (*n* = 6). (D) Representative images of liver and kidney by H&E staining at 12 hours post-infection. Bar: 50 μm. (E) Representative contour plots of γδT17 (CD3^+^ TCRγ/δ^+^ IL-17A^+^) cells in liver at 12 hours post-infection. (F) Scatter plots shown percentage of IL-17A^+^ in γδ T cells in liver at 12 hours post-infection (*n* = 5). (G) Representative contour plots of neutrophils in liver at 12 hours post-infection. (H) Scatter plots shown absolute number of neutrophils in liver at 12 hours post-infection (*n* = 5). Data are presented as means ± SD. *p < 0.05; **p < 0.01; ***p < 0.001; ns, not significant; one-way ANOVA with Tukey’s for multiple comparisons.

From the feces of SPF mice, we isolated 10 strains of commensal bacteria, including a strain identified as *L. reuteri* WX25. Given that *L. reuteri* is a Gram-positive bacterium and was significantly depleted by antibiotics, we hypothesized that it might contribute to host defense. To test this, mice pre-treated with Van+Amp received a 10-day supplementation with *L. reuteri* WX25 prior to *S. aureus i.v*. infection (Figure 6A). *L. reuteri* WX25 supplementation improved survival compared with the Van+Amp group (*p* = 0.0745), and significantly reduced bacterial burdens in the liver. There was no significant difference between *L. reuteri* WX25 and FMT administration (Figure 6B,C). The greater severity of histopathological damage was observed in Van+Amp-treated mice compared with No ABX and *L. reuteri* WX25-treated mice (Figure 6D). Furthermore, *L. reuteri* WX25-treated mice exhibited significantly higher hepatic γδT17 cell and neutrophil populations compared to Van+Amp-treated mice at 12 hours post-infection (Figure 6E–H). These data indicated that commensal *L. reuteri* activates the hepatic γδT17-neutrophil axis promote host defense against systemic *S. aureus* infection.

### Microbiota-derived indole metabolites promote host defense against *S.* *aureus* systemic involving the γδT17/neutrophil axis

**Figure 7.**
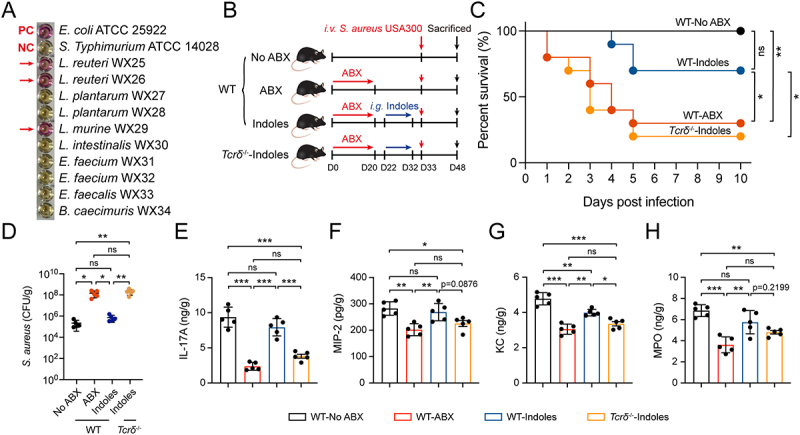
Microbiota-derived indole metabolites promoted host defense against *S. aureus* systemic infection, involving the γδT17/neutrophil axis. (A) Commensal bacteria isolated from the mouse intestine showed colorimetric evidence of indole metabolites production, as determined by the Kovac’s reagent method. (B) Schematic illustration for the gut microbiota elimination and potential effector metabolites treatment. After antibiotic treatment, mice were treated with indole derivatives (IAld+IAA+IPA) by oral gavage (*i.g*.) for 10 days. Subsequently, all mice were challenged *i.v*. with *S. aureus* USA300. (C) Survival curves of No ABX, Van+Amp, FMT, *L. reuteri* WX25 mice after *i.v*. challenged with *S. aureus* (*n* = 10). Statistical significance was determined by the log-rank (mantel-cox) test. (D) Bacterial burden in livers from No ABX, Van+Amp, FMT, *L. reuteri* WX25 mice at 12 hours post-infection (*n* = 5). (E-H) Hepatic IL-17A, MIP-2, KC, and MPO levels in WT-No ABX, WT-ABX, WT-Indoles, and *Tcrδ^−/−^* mice were measured by ELISA at 12 hours post-infection (*n* = 5). Data are presented as means ± SD. *p < 0.05; **p < 0.01; ***p < 0.001; ns, not significant; one-way ANOVA with Tukey’s for multiple comparisons.

While gut microbiota remain localized to the intestines, accumulating evidence indicates that their metabolites can reach distal organs including liver through circulatory pathways. Of particular interest are indole derivatives produced by *L. reuteri* species, which have been shown to exert critical roles in immune-modulatory functions [40–43]. The ability of commensal bacteria, previously isolated from mouse intestines, to produce indole metabolites was assessed using Kovac’s reagent. The results showed that *L. reuteri* WX25, WX26, and *L. intestinalis* WX30 produced strong colorimetric reactions similar to the positive control *E. coli* ATCC 25,922 (Figure 7A). Given the unique role of indole metabolites in mediating interactions between commensal bacteria and the immune system, we hypothesized that these microbial metabolites may influence neutrophil infiltration within the liver microcirculation. To address the role of microbiota-derived indole metabolites in the gut-liver axis, ABX pre-treated mice received daily oral gavage of a mixture of IAld, IAA and IPA for 10 consecutive days prior to *S. aureus i.v*. infection (Figure 7B). The results showed that administration of indole metabolites rescued the survival rate of ABX-treated mice; however, the protective effect was abolished in *Tcrδ*^−/−^ mice (Figure 7C). Additionally, mice supplemented with indole metabolites exhibited significantly lower bacterial loads in the liver during the early stages of infection compared to WT-ABX and *Tcrδ*^−/−^-Indole groups (Figure 7D). The levels of IL-17A, MIP-2, KC, and MPO in the liver were significantly higher in WT-indole group than in the WT-ABX group (Figure 7E–H). These data indicate that microbiota-derived indole metabolites promote host defense against *S. aureus* systemic involving the γδT17/neutrophil axis.

## Discussion

In this study, we demonstrate that the gut microbiota contributes to host defense by promoting neutrophil recruitment to the liver during early stages of systemic *S. aureus* infection. Gut microbiota activated hepatic γδ T cells to promote rapid IL-17A production, neutrophil trafficking and pathogens clearance in the early stages of infection. Disruption of this gut–liver axis, either by antibiotic depletion of the gut microbiota or impairment of the γδ T cell-IL-17A signaling axis, results in a failure to clear pathogens from the blood and leads to disseminated infections. Notably, *L. reuteri* was identified as a key commensal bacterium capable of activating this hepatic γδT17-neutrophil axis and restoring host defense in ABX-treated mice.

The role of the gut microbiota in extra-intestinal immunity is increasingly recognized, but its mechanistic contributions to systemic bacterial clearance remain incompletely defined. Our study specifically elucidated that antibiotic-induced dysbiosis severely compromises hepatic immunity during *S. aureus* bloodstream infection. These observations align with clinical evidence linking gut dysbiosis to increased susceptibility to sepsis and hospital-acquired infections [10–13]. Patients receiving broad-spectrum antibiotics, particularly in ICU, are at an elevated risk of such infections, likely due to microbiota disruption [44]. This disruption not only promotes the overgrowth of opportunistic pathogens like *Staphylococcus aureus*, *Candida albicans* and *Clostridium difficile*, but also weakens systemic immune defenses, impairing the host’s ability to control disseminated infections [45,46]. These studies highlight the importance in the ICU of optimizing antibiotic utilization – considering both timing and duration – to balance effective infection treatment against the disruption of the gut microbiota.

Our research indicated that bacterial accumulation predominantly occurs in the liver during early-stage infection. Previously researches have emphasized that the critical role of neutrophil recruitment to the liver during systemic infections and sepsis in providing intravascular defense against pathogen dissemination through the bloodstream [18,47,48]. These recruited immune cells collaborate with Kupper cells, the resident liver macrophages, to establish a protective intravascular barrier. This cooperative mechanism effectively captures and eliminates circulating pathogens, including bacteria translocating from the gut [49,50]. The strategic positioning of neutrophils within the liver’s sinusoidal network facilitates their surveillance function and enables the deployment of neutrophil extracellular traps (NETs), further enhancing the filtration and clearance of blood-borne pathogens [51,52]. Our finding reveal that gut microbiota orchestrates neutrophils homing to the liver adds to a growing armamentarium of microbiome–neutrophil interactions that are fundamental to effective host defense. In addition, our findings indicate that the depletion of gut microbiota leads to an increased bacterial burden of *Staphylococcus aureus* in the kidneys. Furthermore, this phenomenon exacerbates liver abscess formation in the later stages of infection. A plausible explanation for this observation is that when intestinal barrier function is compromised, a significant influx of *S. aureus* enters the bloodstream. Should the liver’s capacity to clear these bacteria become saturated or impaired, *S. aureus* will “spill over” into the systemic circulation, leading to bacteremia. Within the systemic bloodstream, *S. aureus* exhibits an inherent tropism for the kidneys, readily adhering to endothelial cells and the glomerular basement membrane. Coupled with the kidney’s high blood flow, which renders it highly susceptible to blood-borne pathogen invasion, this provides an opportunity for *S. aureus* to colonize and proliferate within the glomeruli, renal tubules, and interstitial spaces, subsequently forming microabscesses.

Furthermore, our study identifies IL-17A is a pivotal cytokine for translating the effects of the microbiota into increased resistance to systemic *S. aureus* infection. Following *S. aureus* infection, a response significantly attenuated by antibiotic treatment. IL-17A is known to regulate the production of chemokines, such as KC/CXCL1 and MIP-2/CXCL2, essential for neutrophil recruitment [53]. Gut-derived bacterial products promote bone marrow myelopoiesis via a TLR4/MyD88 feedback loop involving intestinal innate lymphoid cells and the production of IL-17A and granulocyte-colony stimulating factor (G-CSF) [5,7,8,20]. This mechanism allows gut bacteria to regulate the systemic availability of neutrophils (and monocytes) for combating pathogenic threat. Our data demonstrate that the gut microbiota promotes the production of these chemokines, strengthening the link between the microbiota, IL-17A, and neutrophil mobilization to the liver during infection.

In the liver, several cellular sources of IL-17A contribute to enhanced innate hepatic defense, including Th17 cells, γδ T cells, and various innate immune cell populations. Much of the focus on IL-17 production regulation has been on Th17 cells; however, several studies have indicated that γδT17 cells are an important early source of IL-17A in *S. aureus* infection [54,55]. Consistently, our findings indicate that γδ T cells serve as a predominant early source of IL-17A in hepatic immune defense against *S. aureus* infection. This aligns with the rapid kinetics of γδ T cell activation and their capacity for swift IL-17A production, within hours of pathogen exposure [56]. The critical role of hepatic γδ T cells is further evidenced by the heightened susceptibility of *Tcrδ*^−/−^ mice to infection, coupled with the complete rescue of protective immunity through exogenous rIL-17A administration. Moreover, we discovered that disruption of gut microbiota impairs the γδT17-neutrophil axis during infection, which extends and enriches the understanding of the role and regulatory mechanisms of hepatic γδ T cells in modulating local immune responses within the liver.

Remarkably, we found that a single species of *Lactobacillus*, *L. reuteri*, was significantly decreased in ABX-treat mice, and was an effective probiotic monotherapy in preventing systemic *S. aureus* infection. However, monocolonization with *L. reuteri* did not completely rescue neutrophils function to the level of observed in SPF mice, and thus, additional microbe-derived signals may contribute to regulating of hepatic defense. While further studies are needed to elucidate the precise mechanisms by which *L. reuteri* exerts its protective effects, our findings suggest that it may act by promoting γδ T cell IL-17A production and subsequent neutrophil recruitment.

In summary, our study provides novel mechanistic insights into how the gut microbiota orchestrates an early hepatic immune defense against systemic *S. aureus* infection. We demonstrate that signals from commensal microorganism prime hepatic γδ T cells to produce IL-17A, which in turn promotes neutrophil recruitment – highlighting a critical gut–liver immune axis. The detailed understanding is especially important considering the clinical context where gut dysbiosis – often induced by antibiotic therapy – significantly increases susceptibility to *S. aureus* infections, particularly among vulnerable populations like ICU patients. Extensive research has shown that critically ill ICU patients frequently experience severe gut dysbiosis, which correlates with higher risks of nosocomial infections, organ failure, and mortality [12,13,57,58]. Our findings implicate impaired liver immune defense mediated by the hepatic γδT17 cell-neutrophil axis may as a mechanism underlying this association between dysbiosis and disseminated infections in the ICU. More importantly, from a translational medicine perspective, our findings suggest that the host defense defects caused by gut dysbiosis can be improved by supplementation with the probiotic *L. reuteri*, which may be a viable precision-medicine strategy. As our understanding of the mechanisms mediating the microbiome-immune crosstalk in systemic host defense deepens, immunomodulation through targeted microbiome interventions (microbial immunotherapy) presents a promising avenue for developing precisely guided interventions to prevent and treat infections.

## Limitations of the study

This study‘s primary focus lies in the early stages of infection. However, the long-term effects of gut microbiota modulation on *S. aureus* infection and the potential development of adaptive immune responses remain areas for further exploration. While our study effectively demonstrates the necessity of the gut microbiota for activating the hepatic γδT17-neutrophil axis, future research could further explore how varying microbial diversity and composition – such as the enrichment of specific commensals or altered community structures – modulate this axis and impact host defense. Moreover, neutrophils collaborate with other immune cells in the liver microcirculation to clear bloodstream pathogens, including KCs, which are themselves functionally modulated by gut microbiota signals including *L. reuteri*. Lastly, although our data suggest a direct effect of *L. reuteri* on hepatic immunity, the precise mechanism by which it activates γδ T cells is still unclear, and indirect effects mediated by other commensal species cannot be excluded.

## Data Availability

The demultiplexed reads for 16S amplicon sequencing data has been deposited at the NCBI Sequence Read Archive (http://ncbi.nlm.nih.gov/sra), and BioProject number was PRJNA1244564. The 16S rRNA sequencing results for strain identification has been deposited at the NCBI Genbank (https://www.ncbi.nlm.nih.gov/nuccore), and GenBank number was PV422614. The data is publicly available on the findings of this study and unrestricted re-use is permitted via open license. This paper does not report original code. The Data generated during the study is available at Mendeley Data (https://data.mendeley.com; doi: 10.17632/w72shkvs2c.2).

## Abbreviations

ABX: Antibiotic-treated
ALT: Alanine aminotransferase
Amp: Ampicillin
ANOVA: One-way analysis of variance
AST: Aspartate aminotransferase
*B.caecimuris*: *Bacteroides caecimuris*
CFUs: Colony forming units
CXCL: Chemokine (C-X-C motif) ligand 1
*E. coli*: *Escherichia coli*
*E. faecalis*: *Enterococcus faecalis*
*E. faecium*: *Enterococcus faecium*
ELISA: Enzyme-linked immunosorbent assay
FCM: Flow cytometry
FMT: Fecal microbiota transplantation
GF: Germ-free
GFP: Green fluorescent protein
H&E: Hematoxylin and eosin
IAA: Indole-3-acetic acid
IAld: Indol-3-aldehyde
ICU: Intensive care unit
*i.g*: Oral gavage
IL-17A: Interleukin-17A
*Il17a*^−/−^ mice: IL-17A knockout mice
IPA: Indole-3-proponic acid
*i.v*: Intravenously
KCs: Kupffer cells
*L. intestinalis*: *Lactobacillus intestinalis*
*L. murine*: *Ligilactobacillus murinus*
*L. plantarum*: *Lactiplantibacillus plantarum*
*L. reuteri*: *Limosilactobacillus reuteri*
LB plate: Luria-Bertani plate
MNCs: Mononuclear cells
MPO: Myeloperoxidase
MRS: Man, Rogosa, and Sharpe
MRSA: Methicillin-resistant *S. aureus*
MSA: Mannitol salt agar
Neo: Neomycin
No ABX: Untreated
PBS: Phosphate-buffered saline
PTT: Peptone-tryptone-tryptophan media
RBC: Red blood cell lysis
rDNA: ribosomal DNA
rIL-17A: Recombinant IL-17A
*S. aureus*: *Staphylococcus aureus*
*S. typhimurium*: *Salmonella enterica* serovar *Typhimurium*
SPF: Specific pathogen-free
*Tcrδ*^−/−^ mice: γδ T cell-deficient mice
Th17: T helper cell 17
TLR: Toll-like receptor
Van: Vancomycin
WT: Wild type

## Inclusion and diversity

We support inclusive, diverse, and equitable conduct if research. We avoided “helicopter science” practices by including the participating local contributors from the region where we conducted the research as authors on the paper.

## References

[1] Turner NA, Sharma-Kuinkel BK, Maskarinec SA, et al. Methicillin-resistant staphylococcus aureus: an overview of basic and clinical research. Nat Rev Microbiol. 2019;17(4):203–18. doi: 10.1038/s41579-018-0147-4 30737488 PMC6939889

[2] van Hal SJ, Jensen SO, Vaska VL, et al. Predictors of mortality in Staphylococcus aureus bacteremia. Clin Microbiol Rev. 2012;25(2):362–386. doi: 10.1128/CMR.05022-11 22491776 PMC3346297

[3] Thaiss CA, Zmora N, Levy M, et al. The microbiome and innate immunity. Nature. 2016;535(7610):65–74. doi: 10.1038/nature18847 27383981

[4] Honda K, Littman DR. The microbiota in adaptive immune homeostasis and disease. Nature. 2016;535(7610):75–84. doi: 10.1038/nature18848 27383982

[5] Khosravi A, Yáñez A, Price J, et al. Gut microbiota promote hematopoiesis to control bacterial infection. Cell Host Microbe. 2014;15(3):374–381. doi: 10.1016/j.chom.2014.02.006 24629343 PMC4144825

[6] Erny D, Hrabě de Angelis AL, Jaitin D, et al. Host microbiota constantly control maturation and function of microglia in the CNS. Nat Neurosci. 2015;18(7):965–977. doi: 10.1038/nn.4030 26030851 PMC5528863

[7] Deshmukh HS, Liu Y, Menkiti OR, et al. The microbiota regulates neutrophil homeostasis and host resistance to Escherichia coli K1 sepsis in neonatal mice. Nat Med. 2014;20(5):524–530. doi: 10.1038/nm.3542 24747744 PMC4016187

[8] Zhang D, Chen G, Manwani D, et al. Neutrophil ageing is regulated by the microbiome. Nature. 2015;525(7570):528–532. doi: 10.1038/nature15367 26374999 PMC4712631

[9] Clarke TB, Davis KM, Lysenko ES, et al. Recognition of peptidoglycan from the microbiota by Nod1 enhances systemic innate immunity. Nat Med. 2010;16(2):228–231. doi: 10.1038/nm.2087 20081863 PMC4497535

[10] Liu Z, Li N, Fang H, et al. Enteric dysbiosis is associated with sepsis in patients. The FASEB J. 2019;33(11):12299–12310. doi: 10.1096/fj.201900398RR 31465241 PMC6902702

[11] Prescott HC, Dickson RP, Rogers MAM, et al. Hospitalization type and subsequent severe sepsis. Am J Respir Crit Care Med. 2015;192(5):581–588. doi: 10.1164/rccm.201503-0483OC 26016947 PMC4595694

[12] Lamarche D, Johnstone J, Zytaruk N, et al. Microbial dysbiosis and mortality during mechanical ventilation: a prospective observational study. Respir Res. 2018;19(1):245. doi: 10.1186/s12931-018-0950-5 30526610 PMC6286574

[13] Freedberg DE, Zhou MJ, Cohen ME, et al. Pathogen colonization of the gastrointestinal microbiome at intensive care unit admission and risk for subsequent death or infection. Intensive Care Med. 2018;44(8):1203–1211. doi: 10.1007/s00134-018-5268-8 29936583 PMC6309661

[14] Lankelma JM, Birnie E, Weehuizen TAF, et al. The gut microbiota as a modulator of innate immunity during melioidosis. PLOS Negl Trop Dis. 2017;11(4):e0005548. doi: 10.1371/journal.pntd.0005548 28422970 PMC5411098

[15] Schuijt TJ, Lankelma JM, Scicluna BP, et al. The gut microbiota plays a protective role in the host defence against pneumococcal pneumonia. Gut. 2016;65(4):575–583. doi: 10.1136/gutjnl-2015-309728 26511795 PMC4819612

[16] Gray J, Oehrle K, Worthen G, et al. Intestinal commensal bacteria mediate lung mucosal immunity and promote resistance of newborn mice to infection. Sci Transl Med. 2017;9(376). doi: 10.1126/scitranslmed.aaf9412 PMC5880204 28179507

[17] Hickey MJ, Kubes P. Intravascular immunity: the host–pathogen encounter in blood vessels. Nat Rev Immunol. 2009;9(5):364–375. doi: 10.1038/nri2532 19390567

[18] Zucoloto AZ, Schlechte J, Ignacio A, et al. Vascular traffic control of neutrophil recruitment to the liver by microbiota-endothelium crosstalk. Cell Rep. 2023;42(5):112507. doi: 10.1016/j.celrep.2023.112507 37195866

[19] Schlechte J, Skalosky I, Geuking MB, et al. Long-distance relationships - regulation of systemic host defense against infections by the gut microbiota. Mucosal Immunol. 2022;15(5):809–818. doi: 10.1038/s41385-022-00539-2 35732817

[20] Balmer ML, Schürch CM, Saito Y, et al. Microbiota-derived compounds drive steady-state granulopoiesis via MyD88/TICAM signaling. J Immunol. 2014;193(10):5273–5283. doi: 10.4049/jimmunol.1400762 25305320

[21] Fagundes CT, Amaral FA, Vieira AT, et al. Transient TLR activation restores inflammatory response and ability to control pulmonary bacterial infection in germfree mice. J Immunol. 2012;188(3):1411–1420. doi: 10.4049/jimmunol.1101682 22210917

[22] Kanther M, Tomkovich S, Xiaolun S, et al. Commensal microbiota stimulate systemic neutrophil migration through induction of serum amyloid A. Cell Microbiol. 2014;16(7):1053–1067. doi: 10.1111/cmi.12257 24373309 PMC4364439

[23] Karmarkar D, Rock KL. Microbiota signalling through MyD88 is necessary for a systemic neutrophilic inflammatory response. Immunology. 2013;140(4):483–492. doi: 10.1111/imm.12159 23909393 PMC3839652

[24] Hammerich L, Tacke F. Role of gamma-delta T cells in liver inflammation and fibrosis. World J Gastrointest Pathophysiol. 2014;5(2):107–113. doi: 10.4291/wjgp.v5.i2.107 24891982 PMC4025070

[25] Ribot JC, Lopes N, Silva-Santos B. γδ T cells in tissue physiology and surveillance. Nat Rev Immunol. 2021;21(4):221–232. doi: 10.1038/s41577-020-00452-4 33057185

[26] Papotto PH, Yilmaz B, Silva-Santos B. Crosstalk between γδ T cells and the microbiota. Nat Microbiol. 2021;6(9):1110–1117. doi: 10.1038/s41564-021-00948-2 34341528

[27] Marchitto MC, Dillen CA, Liu H, et al. Clonal Vγ6 + Vδ4 + T cells promote IL-17–mediated immunity against Staphylococcus aureus skin infection. Proc Natl Acad Sci USA. 2019;116(22):10917–10926. doi: 10.1073/pnas.1818256116 31088972 PMC6561199

[28] Cho JS, Pietras EM, Garcia NC, et al. Il-17 is essential for host defense against cutaneous Staphylococcus aureus infection in mice. J Clin Invest. 2010;120(5):1762–1773. doi: 10.1172/JCI40891 20364087 PMC2860944

[29] Pan N, Xiu L, Xu Y, et al. Mammary γδ T cells promote IL-17A-mediated immunity against Staphylococcus aureus-induced mastitis in a microbiota-dependent manner. iScience. 2023;26(12):108453. doi: 10.1016/j.isci.2023.108453 38034361 PMC10687336

[30] Li F, Hao X, Chen Y, et al. The microbiota maintain homeostasis of liver-resident γδt-17 cells in a lipid antigen/CD1d-dependent manner. Nat Commun. 2017;8(1):13839. doi: 10.1038/ncomms13839 PMC5227332 28067223

[31] Jin C, Lagoudas GK, Zhao C, et al. Commensal microbiota promote lung cancer development via γδ T cells. Cell. 2019;176(5):998–1013.e16. doi: 10.1016/j.cell.2018.12.040 30712876 PMC6691977

[32] Dupraz L, Magniez A, Rolhion N, et al. Gut microbiota-derived short-chain fatty acids regulate IL-17 production by mouse and human intestinal γδ T cells. Cell Rep. 2021;36(1):109332. doi: 10.1016/j.celrep.2021.109332 34233192

[33] McDonald B, Zucoloto AZ, Yu I-L, et al. Programing of an intravascular immune firewall by the gut microbiota protects against pathogen dissemination during infection. Cell Host Microbe. 2020;28(5):660–668.e4. doi: 10.1016/j.chom.2020.07.014 32810440

[34] Hu X, Guo J, Zhao C, et al. The gut microbiota contributes to the development of Staphylococcus aureus-induced mastitis in mice. Isme J. 2020;14(7):1897–1910. doi: 10.1038/s41396-020-0651-1 32341472 PMC7305118

[35] Brown RL, Sequeira RP, Clarke TB. The microbiota protects against respiratory infection via GM-CSF signaling. Nat Commun. 2017;8(1):1512. doi: 10.1038/s41467-017-01803-x 29142211 PMC5688119

[36] Surewaard BG, Deniset JF, Zemp FJ, et al. Identification and treatment of the Staphylococcus aureus reservoir in vivo. J Exp Med. 2016;213(7):1141–1151. doi: 10.1084/jem.20160334 27325887 PMC4925027

[37] Nguyen CT, Furuya H, Das D, et al. Peripheral γδ T cells regulate neutrophil expansion and recruitment in experimental psoriatic arthritis. Arthritis Rheumatol. 2022;74(9):1524–1534. doi: 10.1002/art.42124 35320625 PMC9427669

[38] Yang D, Chen X, Wang J, et al. Dysregulated lung commensal bacteria drive interleukin-17B production to promote pulmonary fibrosis through their outer membrane vesicles. Immunity. 2019;50(3):692–706.e7. doi: 10.1016/j.immuni.2019.02.001 30824326

[39] Cervantes-Barragan L, Chai JN, Tianero MD, et al. Lactobacillus reuteri induces gut intraepithelial CD4 + CD8αα + T cells. Science. 2017;357(6353):806–810. doi: 10.1126/science.aah5825 28775213 PMC5687812

[40] Bender MJ, McPherson AC, Phelps CM, et al. Dietary tryptophan metabolite released by intratumoral Lactobacillus reuteri facilitates immune checkpoint inhibitor treatment. Cell. 2023;186(9):1846–1862.e26. doi: 10.1016/j.cell.2023.03.011 37028428 PMC10148916

[41] Chi YY, Xiang J-Y, Li H-M, et al. Schisandra chinensis polysaccharide prevents alcohol-associated liver disease in mice by modulating the gut microbiota-tryptophan metabolism-AHR pathway axis. Int J Biol Macromol. 2024;282(Pt 2):136843. doi: 10.1016/j.ijbiomac.2024.136843 39461640

[42] Fang H, Fang M, Wang Y, et al. Indole-3-propionic acid as a potential therapeutic agent for sepsis-induced gut microbiota disturbance. Microbiol Spectr. 2022;10(3):e0012522. doi: 10.1128/spectrum.00125-22 35658593 PMC9241804

[43] Zhao C, Hu X, Bao L, et al. Aryl hydrocarbon receptor activation by Lactobacillus reuteri tryptophan metabolism alleviates Escherichia coli-induced mastitis in mice. PLOS Pathog. 2021;17(7):e1009774. doi: 10.1371/journal.ppat.1009774 34297785 PMC8336809

[44] Kollef MH, Shorr AF, Bassetti M, et al. Timing of antibiotic therapy in the ICU. Crit Care. 2021;25(1):360. doi: 10.1186/s13054-021-03787-z 34654462 PMC8518273

[45] Smith AB, Jenior ML, Keenan O, et al. Enterococci enhance Clostridioides difficile pathogenesis. Nature. 2022;611(7937):780–786. doi: 10.1038/s41586-022-05438-x 36385534 PMC9691601

[46] Drummond RA, Desai JV, Ricotta EE, et al. Long-term antibiotic exposure promotes mortality after systemic fungal infection by driving lymphocyte dysfunction and systemic escape of commensal bacteria. Cell Host Microbe. 2022;30(7):1020–1033.e6. doi: 10.1016/j.chom.2022.04.013 35568028 PMC9283303

[47] Paudel S, Baral P, Ghimire L, et al. Cxcl1 regulates neutrophil homeostasis in pneumonia-derived sepsis caused by Streptococcus pneumoniae serotype 3. Blood. 2019;133(12):1335–1345. doi: 10.1182/blood-2018-10-878082 30723078 PMC6428667

[48] Hamada S, Umemura M, Shiono T, et al. Il-17a produced by γδ T cells plays a critical role in innate immunity against Listeria monocytogenes infection in the liver. J Immunol. 2008;181(5):3456–3463. doi: 10.4049/jimmunol.181.5.3456 18714018 PMC2859669

[49] Kubes P, Jenne C. Immune responses in the liver. Annu Rev Immunol. 2018;36(1):247–277. doi: 10.1146/annurev-immunol-051116-052415 29328785

[50] Balmer ML, Slack E, de Gottardi A, et al. The liver may act as a firewall mediating mutualism between the host and its gut commensal microbiota. Sci Transl Med. 2014;6(237):237ra66. doi: 10.1126/scitranslmed.3008618 24848256

[51] McDonald B, Urrutia R, Yipp B, et al. Intravascular neutrophil extracellular traps capture bacteria from the bloodstream during sepsis. Cell Host Microbe. 2012;12(3):324–333. doi: 10.1016/j.chom.2012.06.011 22980329

[52] Clark SR, Ma AC, Tavener SA, et al. Platelet TLR4 activates neutrophil extracellular traps to ensnare bacteria in septic blood. Nat Med. 2007;13(4):463–469. doi: 10.1038/nm1565 17384648

[53] Luo CJ, Luo F, Zhang L, et al. Knockout of interleukin-17A protects against sepsis-associated acute kidney injury. Ann Intensive Care. 2016;6(1):56. doi: 10.1186/s13613-016-0157-1 27334720 PMC4917508

[54] Sutton CE, Lalor SJ, Sweeney CM, et al. Interleukin-1 and IL-23 induce innate IL-17 production from γδ T cells, amplifying Th17 responses and autoimmunity. Immunity. 2009;31(2):331–341. doi: 10.1016/j.immuni.2009.08.001 19682929

[55] Edwards SC, Sutton CE, Ladell K, et al. A population of proinflammatory T cells coexpresses αβ and γδ T cell receptors in mice and humans. J Exp Med. 2020;217(5). doi: 10.1084/jem.20190834 PMC7201916 32106283

[56] Mills KHG. Il-17 and il-17-producing cells in protection versus pathology. Nat Rev Immunol. 2023;23(1):38–54. doi: 10.1038/s41577-022-00746-9 35790881 PMC9255545

[57] Ojima M, Motooka D, Shimizu K, et al. Metagenomic analysis reveals dynamic changes of whole gut microbiota in the acute phase of intensive care unit patients. Dig Dis Sci. 2016;61(6):1628–1634. doi: 10.1007/s10620-015-4011-3 26715502 PMC4875048

[58] Xu Y, Xiang Z, Alnaggar M, et al. Allogeneic Vγ9Vδ2 t-cell immunotherapy exhibits promising clinical safety and prolongs the survival of patients with late-stage lung or liver cancer. Cell Mol Immunol. 2021;18(2):427–439. doi: 10.1038/s41423-020-0515-7 32939032 PMC8027668

